# Sexual attraction-based disparities in adolescent mental health: The role of school norms

**DOI:** 10.1093/eurpub/ckac129.677

**Published:** 2022-10-25

**Authors:** WJ Kiekens, L Baams, GWJM Stevens

**Affiliations:** 1 Department of Sociology, ICS, University of Groningen, Groningen, Netherlands; 2 Department of Pedagogy and Educational Sciences, University of Groningen, Groningen, Netherlands; 3 Department of Interdisciplinary Social Sciences, Utrecht University, Utrecht, Netherlands

## Abstract

**Purpose:**

Few researchers have explained disparities in mental health between sexual minority and heterosexual adolescents by focusing on structural forms of stigma as, for instance, heterosexist school or classroom norms. Addressing this gap, our paper aimed to study disparities in life satisfaction, psychosomatic complaints, and emotional problems between sexual minority and heterosexual adolescents and examine the moderating role of heterosexist norms in the classroom and school.

**Methods:**

We used data from the 2013 and 2017 Dutch Health and Behaviour in School-Aged Children (HBSC) study (N = 12,756; M age = 14.02; SD = 1.54). Separate multi-level analyses for life satisfaction, psychosomatic complaints, and emotional problems were conducted in which cross-level interaction effects between sexual attraction and school and classroom-level heterosexist norms were estimated.

**Results:**

Same-sex attracted, both-sex attracted, and adolescents unsure about their sexual attraction reported lower life satisfaction, more psychosomatic complaints (not for unsure adolescents), and more emotional problems than their other-sex attracted peers. Stronger school-level heterosexist norms were associated with higher life satisfaction and fewer psychosomatic complaints and fewer emotional problems. Stronger classroom-level heterosexist norms were associated with less emotional problems. Few moderating effects of classroom and school-level heterosexist norms were found. Contrary to expectations, disparities in life satisfaction between same-sex attracted and other-sex attracted adolescents decreased when classroom-level heterosexist norms were stronger.

**Conclusions:**

Although our findings suggest pressing health disparities between heterosexual and sexual minority adolescents, heterosexist norms at the school- and classroom-level hardly contributed to these health disparities.

